# Gene family structure, expression and functional analysis of HD-Zip III genes in angiosperm and gymnosperm forest trees

**DOI:** 10.1186/1471-2229-10-273

**Published:** 2010-12-11

**Authors:** Caroline L Côté, Francis Boileau, Vicky Roy, Mario Ouellet, Caroline Levasseur, Marie-Josée Morency, Janice EK Cooke, Armand Séguin, John J MacKay

**Affiliations:** 1 Département des Sciences du Bois et de la Forêt, Université Laval, 2405 rue de la Terrasse, Québec, QC, G1V 0A6, Canada; 2 Laurentian Forestry Centre, 1055 rue du P.E.P.S., Québec, QC, G1V 4C7, Canada; 3 BEI, Joint BioEnergy Institute, 5885 Hollis St, 4th floor, Emeryville, CA 94608, USA; 4 Department of Biological Sciences, University of Alberta, Edmonton, AB, T6G 2E9, USA

## Abstract

**Background:**

Class III Homeodomain Leucine Zipper (HD-Zip III) proteins have been implicated in the regulation of cambium identity, as well as primary and secondary vascular differentiation and patterning in herbaceous plants. They have been proposed to regulate wood formation but relatively little evidence is available to validate such a role. We characterised and compared HD-Zip III gene family in an angiosperm tree, *Populus *spp. (poplar), and the gymnosperm *Picea glauca *(white spruce), representing two highly evolutionarily divergent groups.

**Results:**

Full-length cDNA sequences were isolated from poplar and white spruce. Phylogenetic reconstruction indicated that some of the gymnosperm sequences were derived from lineages that diverged earlier than angiosperm sequences, and seem to have been lost in angiosperm lineages. Transcript accumulation profiles were assessed by RT-qPCR on tissue panels from both species and in poplar trees in response to an inhibitor of polar auxin transport. The overall transcript profiles HD-Zip III complexes in white spruce and poplar exhibited substantial differences, reflecting their evolutionary history. Furthermore, two poplar sequences homologous to HD-Zip III genes involved in xylem development in *Arabidopsis *and *Zinnia *were over-expressed in poplar plants. *PtaHB1 *over-expression produced noticeable effects on petiole and primary shoot fibre development, suggesting that *PtaHB1 *is involved in primary xylem development. We also obtained evidence indicating that expression of *PtaHB1 *affected the transcriptome by altering the accumulation of 48 distinct transcripts, many of which are predicted to be involved in growth and cell wall synthesis. Most of them were down-regulated, as was the case for several of the poplar HD-Zip III sequences. No visible physiological effect of over-expression was observed on *PtaHB7 *transgenic trees, suggesting that *PtaHB1 *and *PtaHB7 *likely have distinct roles in tree development, which is in agreement with the functions that have been assigned to close homologs in herbaceous plants.

**Conclusions:**

This study provides an overview of HD-zip III genes related to woody plant development and identifies sequences putatively involved in secondary vascular growth in angiosperms and in gymnosperms. These gene sequences are candidate regulators of wood formation and could be a source of molecular markers for tree breeding related to wood properties.

## Background

The differentiation of vascular tissues is an intensively studied aspect of plant development. Part of this interest is driven by the economic importance of xylem as a major constituent of forage crops, wood, and lignocellulosic biomass for transport fuels. Xylem is characterised by highly specialised and easily identifiable water-conducting cell types, i.e. tracheids in gymnosperms and tracheary elements (TEs) in angiosperms. Xylem also contributes to the physical support of plant structures, which is imparted by either fibres (in angiosperms) or tracheids. Primary xylem arises through the differentiation of pro-vascular cells near the apical meristem and secondary xylem differentiates from fusiform initials in the cambial zone [1]. Environmental conditions and developmental state modulate xylem composition and properties [2], as well as cell characteristics [3], through the action of growth regulators such as auxin, ethylene, and gibberellins, together with regulatory proteins such as transcription factors.

Insights into the regulatory components of xylem development, including transcriptional regulators, have been derived from functional analyses in the herbaceous model plants *Arabidopsis thaliana *(L.) Heynh., *Zinnia elegans *(Jacq.), and *Oryza sativa *(L.) [4,5]. HOMEO-DOMAIN LEUCINE ZIPPER CLASS III (HD-Zip III) proteins represent a group of transcription factors that have been extensively implicated in the regulation of primary and secondary vascular tissue pattern formation, as well as lateral organ and cambial polarity in herbaceous annual plants. It stands to reason that HD-Zip IIIs may also play key roles in secondary vascular growth and wood formation in perennials including shrubs and trees, but there is relatively little evidence to elucidate such a role, except for the report by Ko *et al*. (2006) [6].

There are several different classes of plant homeobox genes [7]. One of the major groups of these genes is HD-Zip, which is divided into classes I to IV. Both the DNA-binding Homeodomain (HD) and the basic leucine zipper domain (bZIP), the latter of which has protein dimerization properties [8], are conserved in all four classes. Members of the HD-Zip III and IV classes also share a steroidogenic, acute regulatory protein-related domain associated with the lipid-Transfer (START) domain [9]. In addition, class III HD-Zips have a characteristic C-terminal MEKHLA domain that shares significant similarity with the PAS domain, reported to dimerize with the AP2 domain of the transcription factor DRN/ESR-1 [10] involved in embryo patterning and auxin transport [11].

Five different HD-Zip III proteins have been functionally characterised by different approaches in *A. thaliana*. They include *Revoluta *(*REV/IFL-1/AVB-1*), *Phabulosa *(*phb/AtHB-14*), *Phavoluta *(*phv/AthHB-9*), *Corona *(*cna/AtHB-15*) and *AtHB-8*. Arabidopsis *REVOLUTA *(*rev*) mutants have altered interfascicular fibre development and impaired auxin polar transport [12,13]. Over-expression of *REV *in *Arabidopsis *resulted in weakly radialized vascular bundles, and altered leaf, stem and carpel organ abaxial, adaxial pattern polarity. Over-expression of the *Z. elegans ZeHB-12*, a homologue of *REV*, led to an increased number of xylem precursor cells and the accumulation of a variety of transcripts, including brassinosteroid-related sequences and vascular preferential transcripts in *Zinnia *[14]. Analyses of double *phb*:*phv *mutants showed that the two genes share redundant functions both in establishing organ polarity and in vascular development [15]. In Arabidopsis, *AtHB-8 *is an early marker for procambial development, vein patterning, and differentiation [16]. Its over-expression caused ectopic proliferation of xylem cells and precocious initiation of secondary growth; however, the *Athb-8 *loss-of-function mutant had no obvious vascular phenotype [17]. In contrast, *cna *mutants and antisense plants have increased vascular tissues and defects in organ polarity [18], while *CNA *over-expression leads to smaller vascular bundles, indicating that it likely acts as a negative regulator of procambial cell identity or proliferation. Transcript accumulation in a few HD-Zip III sequences is regulated by auxin (specifically *AtHB-8*) [16] and brassinosteroids [12]. Post-transcriptional gene silencing by microRNAs is highly conserved in plants and specifically targets all of the HD-Zip III genes through the binding of mir165/166 [19].

Functional analyses of HD-Zip III genes in herbaceous plants, including *A*. *thaliana *and *Z. elegans*, have provided a useful template against which similar functions regulating secondary vascular growth can be investigated in woody plants (shrubs, trees) [20]. As genetic selection and breeding activities in trees are being expanded to include genetic mapping and molecular markers, candidate genes like HD-Zip III are considered as potential markers which could be associated with wood properties. In this context, the aim of this study was to characterise the HD-Zip III transcription factor family and assess potential involvement in vascular development of trees. Previous reports [21,22] have provided indications that the number of HD-Zip III genes and gene family structure may vary between species, especially between angiosperms and gymnosperms. We evaluated and compared gene family structure in poplars (*Populus *spp.) and white spruce *Picea glauca *(Moench) *Voss *with that described for herbaceous annuals to clarify the evolutionary status of HD-zip III in these groups. Transcript profiles were examined across several tissues to assess their putative involvement in secondary vascular growth. In poplar, the accumulation of HD-Zip III gene transcripts was specifically examined in differentiating secondary xylem (2X) in relation to auxin transport, a key driver of tracheary element differentiation [23]. The putative roles of poplar genes *PtaHB1 *and *PtaHB7*from to distinct well characterised subclades with contrasted functions in crops were examined with respect to over-expression effects upon vascular differentiation and RNA transcript profiles.

## Results

### Sequence analysis of HD-Zip III genes from conifer and hardwood trees

Four putative full-length HD-Zip III coding sequences were isolated from *P. glauca *by EST data mining, RT-PCR, and RACE cloning (Rapid Amplification cDNA End) with degenerate primers. Two class-IV sequences from *P. abies *have been previously reported and were denoted *PaHB1 *and *PaHB2 *[24]. Therefore, we designated the sequences that we isolated as *PgHB3 *[25] to *PgHB6 *(Additional file 1 Figure 1 HQ391914 to HQ391917). Predicted amino-acid sequences display the structural features of HD-Zip III, except that *PgHB6 *has a partially degenerated leucine zipper motif.

**Figure 1:**
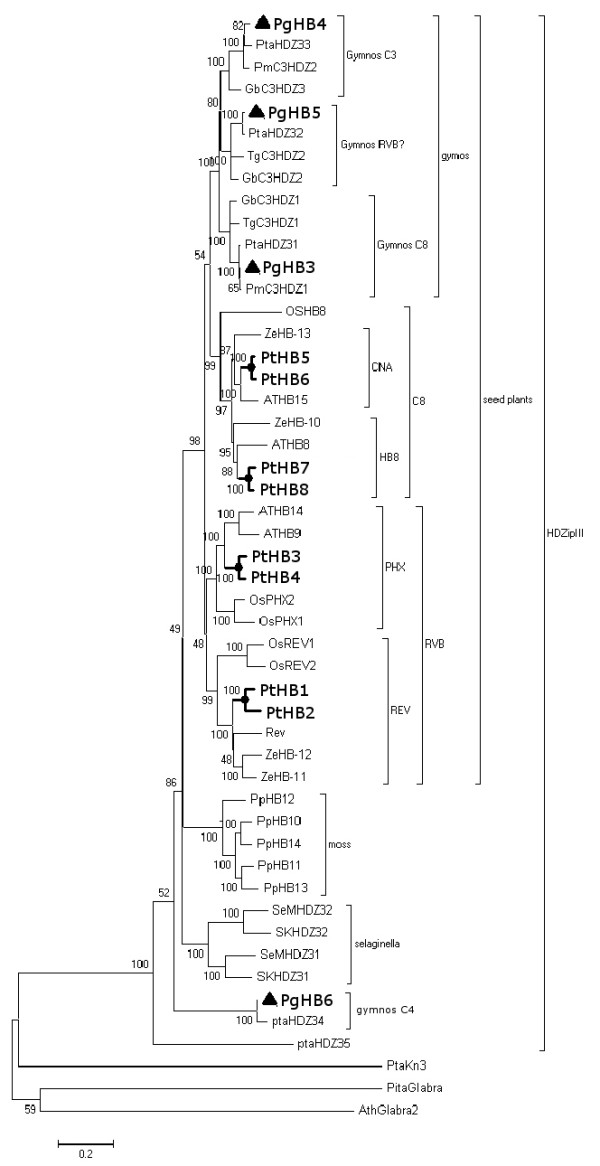
**Cladogram showing the phylogenetic structure of the HD-Zip III gene family**. The Neighbour-Joining (NJ) tree of HD-Zip III sequences was constructed from complete amino acid sequences using, with Poisson correction, 1000 bootstraps and pair-wise deletion parameters. *Populus trichocarpa *(PtHB1 to PtHB8: AY919616.1 to AY919623.1), *Arabidopsis thaliana *(Rev: AK229561.1, ATHB9: NM_102785.4, ATHB14: NM_129025.3, ATHB8: NM_119441.4, ATHB15: NM_104096.1), *Physcomitrella patens *(PpHB10 to PpHB14: DQ6567200.1 to DQ6567204.1), *Picea glauca *(HQ391914 to HQ391917), *Pinus taeda *(PtaHDZ31 to PtaHDZ35: DQ65720.1 to DQ65724.1), *Zinnia elegans *(ZeHB-10: AB084380.1, ZeHB-11:, ZeHB-12:, ZeHB-13:), *Ginkgo biloba *(GbC3HDZ1 ot GbC3HDZ3: DQ385525.1 to DQ385527.1), *Taxus globosa *(TgC3HDZ1: DQ385530.1, TgC3HDZ2: DQ385531.1), *Pseudotsuga menziesii *(PmC3HDZ1: DQ385528.1, PmC3HDZ2: DQ385529.1), *Oryza sativa *(OsHB8: AB374207.1, OsPHX1: AK103283, OsPHX2: AK103284, OsREV1: NM_001057934.1, OsREV2: AK100250.1), *Selaginella kraussiana *(SKHDZ31: DQ657196.1, SKHDZ32: DQ6571971), *Selaginella moellendorffii *(SeMHDZ31: DQ657198.1, SeMHDZ32: DQ657199.1). Black triangles are used for *P. glauca *sequences; bold characters are used for poplar.

The *Populus trichocarpa *genome sequence [26] was reported to contain eight different HD-Zip III sequences, which are designated *HB1 *to *HB8 *[6]. HD-Zip III genes are distributed on seven of the nineteen poplar chromosomes (Additional file 1). We isolated full-length coding cDNA sequences for eight on the putative poplar HD-Zip III genes by RT-PCR, amplification, starting from the *P. trichocarpa *(Torr. & Gray) × *P. deltoides *(W. Bartram) hybrid clone H11-11 and from the *P. tremula *Minch × *P. alba *L. clone 717-1B4. For each of the eight cDNA clones, nearly perfect sequence identities were used to match the cDNA sequences with previously identified ESTs and genes predicted from the poplar genome [6], thus providing evidence that all of the predicted genes are expressed in *Populus *spp.

There are five HD-Zip III genes in the *Arabidopsis *genome belonging to the two major phylogenetic clades RVB and C8, each of which is divided into two subclades [27]. Floyd *et al*. (2006) [21] and Prigge and Clark (2006) [22] conducted phylogenetic investigations that included HD-zip III sequences from diverse plants, along with full-length and partial *Pinus taeda *L. cDNA sequences. They concluded that conifer HD-Zip III genes could be assigned to the two major angiosperm clades of C8 and RVB, but two of the conifer sequences were likely part of gymnosperm-specific clades. In this report, a neighbour-joining (NJ) tree [28] was constructed with complete amino acid sequences from several seed plants, including gymnosperms such as *P. glauca *and *P. taeda*, and angiosperms such as *A. thaliana *and *P*. *trichocarpa*, as well as lower plants such as the moss *Physcomitrella patens *(Hedw.) Bruch & Schimp. The resulting tree topology was consistent with previous reports; however, our data suggest that conifer sequences may in fact be uniquely represented in the C8 clade and absent in the RVB clade (Figure 1). The conifers that we analysed may thus have three C8 members, including sequences previously assigned to the RVB clade. The full-length *P. glauca PgHB6 *and the partial *P. taeda PtaHD-34 *and *PtaHD-35 *fell outside angiosperm clades and formed a monophyletic group, consistent with previous reports [21,22]. Sequence similarity and tree topology clearly grouped the *Populus *sequences as four pairs of closely related paralogues, which is consistent with the ancestral salicoid genome-wide duplication and reorganisation described in modern Salicaceae [29].

### HD-zip III transcripts accumulate during secondary vascular growth in Picea and Populus

**Figure 2:**
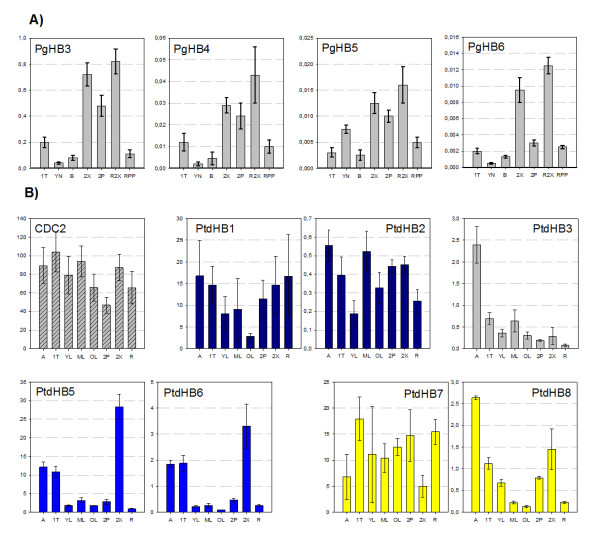
**White spruce and poplar HD-Zip III transcript profiles across several organs and tissues**. Steady-state RNA levels were determined by RT-qPCR with gene-specific primers. The Y-axis is the number of RNA molecules/ng total RNA (determined from a standard curve), which has been normalised based on the transcript accumulation level of a gene. **A) **Mean RNA level in *P. glauca *was analysed in duplicate in two independent biological replicates (one tree per replicate) ± SD (error bar), and normalised based on the transcript accumulation levels of reference gene EF1a. **B) **Mean RNA level in *P. trichocarpa *× *P. deltoides *(clone H11-11) from duplicate analyses of two biological replicates (two trees per replicate) ± SD (error bars), normalised with a CDC_2 _reference gene. The recently duplicated poplar paralogues are colour-matched. The tissue codes (see Methods): shoot apex (A), portion of the main undergoing primary growth (1T), young needles from upper tree crowns (YN, in spruce); young leaves (YF, in poplar); mature leaves (MF); old leaves (OF); bark (B); stem secondary xylem (2X) and phloem (2P); root secondary xylem (R2X); phloem/phelloderm (RPP); and young root tips (R).

Transcript accumulation was profiled in young *P. glauca *and *P. trichocarpa × deltoïdes *trees (refered as *PtdHB*) grown under controlled conditions by using RT-qPCR to compare steady mRNA levels in several organs and tissues (Figure 2). Transcripts of the four spruce sequences accumulated preferentially in the differentiating secondary xylem of stems (2X) and roots (R2X) and gave similar profiles overall (Figure 2A). *PgHB3*, *PgHB4 *and *PgHB5 *RNAs were also abundant in the differentiating secondary phloem (2P), and *PgHB5 *had the highest relative abundance in the young foliage (YL) (Figure 2A). The data suggested that the different transcripts differ substantially in abundance since the normalised number of RNA molecules varied by two orders of magnitude between the highest and lowest RNAs, i.e., *PgHB3 *and *PgHB6*, respectively. The aforementioned data are consistent with putative roles in vascular differentiation, with little indication of diversification between the gene sequences.

Compared with spruce, poplar HD-Zip III genes gave more diversified transcript accumulation profiles across the panel of organs and tissues, even within pairs of closely related paralogues (Figure 2B). The pair *PtdHB1 *and *PtdHB2*, which are close homologues of *REVOLUTA*, gave relatively similar profiles across the panel, except that *PtdHB1 *was less abundant in mature and old leaves than in developing tissues. Furthermore, *PtdHB1 *transcript abundance was two orders of magnitude higher than *PtdHB2*. The pair *PtdHB5 *and *PtdHB6*, closest homologues of *Corona/AtHB-15*, shared similar transcript profiles which varied strongly between the organs surveyed. Both were clearly most abundant in the developing secondary xylem (2X), but also accumulated in the apex and primary stem. On average, *PtdHB5 *was five to ten times more abundant than *PtdHB6*. The pair *PtdHB7 *and *PtdHB8*, which are the closest homologues of *AtHB-8*, gave dissimilar and even opposite transcript profiles. *PtdHB7 *transcripts were abundant in nearly all organs and lowest in the apex (A) and developing secondary xylem (2X), whereas *PtdHB8 *transcripts were most abundant in these same tissues (A, 2X). Transcripts of *PtdHB3*, which was a close homologue of *PHV *and *PHB*, largely accumulated in the apex and to a much lower degree than in other parts of the trees, especially the roots. Data are not reported for *PtdHB4 *because its amplification by RT-qPCR was not strong enough for reliable determinations.

### Over-expression of wild-type PtaHB1 and PtaHB7 genes in transgenic poplars

**Figure 3:**
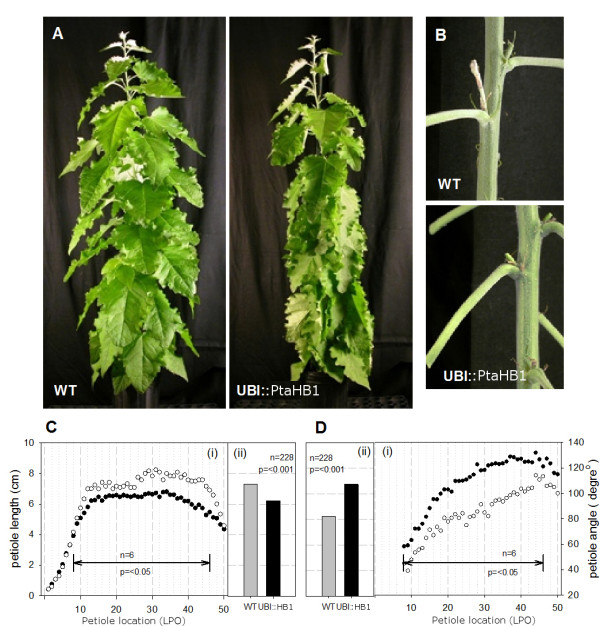
**Altered petiole development in *UBI::PtaHB1 *transgenic poplars**. **(A) **Six-month-old plants of the wild-type (WT) and *UBI::PtaHB1 *(representative of transgenic lines from three independent transformation events). **(B) **Close-up view of mature petioles to show angle relative to the main stem. **(C) **Distribution of petiole lengths (i) from the first fully expanded leaf (inter-node position: LPI 0) to the last healthy leaf (approximately LPI 50). Mean length (ii) was calculated from LPI 8 to LPI 45 (38 internodes, *n *= 228) and Student's *t *test was applied to the data from each internode separately (n = 6 per class; p < 0.05) Histogram bars represent average values (cm). **(D) **Distribution of petiole angle (i) from the first mature leaf below the area of stem elongation (LPI 8) to the last healthy leaf at the bottom (approximately LPI 50). The mean angles (ii) were calculated in the same manner as mean length. Open circles are used for the wild-types and closed circles for the *PtaHB1 *transgenic (**C, D**).

Transgenic poplar trees that overexpressed the complete coding sequence of *PtaHB1 *and *PtaHB7 *were obtained to investigate the potential roles of these HD-Zip III genes in tree development. The hybrid poplar clone INRA-717-1B4 (*P. tremula × P. alba*) was transformed using *Agrobacterium *with either one of the *PtaHB *constructs or an empty vector control (WT). Several hygromycin-resistant and GUS-positive lines were recovered and used to produce viable plants grown to an average height of 1.20 m in the greenhouse. All of the lines had transgene transcript accumulation levels which were significantly above levels detected for the INRA-717 endogene (Table 1). Interestingly, all of the lines overexpressing *PtaHB1 *(*UBI::PtaHB1*) had a visible external phenotype that was not seen in the controls (Figure 3), but no phenotype was observed upon over-expression of *PtaHB7 *(data not show).

**Table 1:** Relative transcript abundance of HD-Zip III gene family members in transgenic poplars.

Transgene construct	Gene	Mean log_2 _ratio	SD	*p*-value
*UBI:PtaHB-1*	PtaHB-1***	**2.7320**	**0.4340**	**<0.001**
	
	PtaHB-2	-0.7020	0.4580	0.0540
	
	PtaHB-3	-0.5330	0.4730	0.1500
	
	PtaHB-5*	**-0.7920**	**0.4960**	**0.0430**
	
	PtaHB-6	-0.5060	0.4840	0.1770
	
	PtaHB-7	-0.4920	0.4490	0.1470
	
	PtaHB-8	-0.5100	0.4600	0.1280

*UBI:PtaHB-7*	PtaHB-1*	**0.5521**	**0.1869**	**0.0318**
	
	PtaHB-2	0.4272	0.3613	0.2761
	
	PtaHB-3*	**0.7675**	**0.1940**	**0.0148**
	
	PtaHB-5*	**0.7263**	**0.1578**	**0.0046**
	
	PtaHB-6	0.6951	0.2727	0.0573
	
	PtaHB-7***	**2.8634**	**0.2689**	**0.0008**
	
	PtaHB-8	0.3194	0.1490	0.0838

Total RNA from the same samples as those used for the microarray profiling: portion of the main undergoing primary growth (IT) for *UBI::PtaHB1; scrapped secondary xylem *(S2X) *for UBI::PtaHB7 *transgenic poplar. *p < 0.05, ***p < 0.001(Student's *t *test, N = 6); SD, one standard deviation.

Further characterisation of the *PtaHB1 *transformed trees showed that *PtaHB1 *transgene transcripts were five to eight times more abundant than the *PtaHB1 *endogene in the controls. The most obvious phenotype in these trees was their drooping leaves. The trees appeared to have a water-stress phenotype (Figure 3A) which was clearly not the case given that they were grown alongside perfectly healthy control trees. Upon closer inspection, it was evident that *PtaHB1 *over-expression resulted in altered petiole development, causing the leaves to hang downward. Other than the petiole, the leaves seemed to develop normally and to be perfectly healthy, with no indications of altered water relations. On average, the transgenic poplars had petioles that were 15% shorter, and the angle between the adaxial side of the leaf and the stem was 30% wider than those of control trees (Figure 3B). The increased angle and decreased length were statistically significant starting at the 10^th ^internode from the apex (where the first internode is the first leaf longer than 1 cm) (*p *< 0.05) (Figure 3C, D).

**Figure 4:**
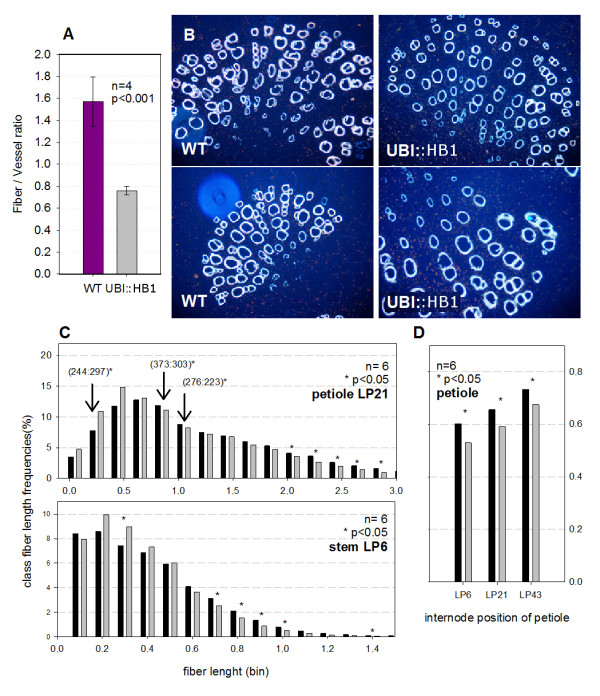
**Altered fibre development in petioles and stems of transgenic *UBI::PtaHB1 *poplars**. **(A) **Mean (± SD) ratio of fibre vessel elements determined from image analyses based on four separate petioles for two transgenic lines carrying the *UBI::HB1 *construct and one wild-type differed significantly according to Student's *t *test (*p *< 0.001). **(B) **Cross-sections of mature petiole (40×, LP 21), observed under UV-illumination to reveal autofluorescence of lignified cell walls of fibre and vessel elements. **(C) **Distribution of fibre lengths in mature petioles (LPI 21) and partially lignified stems (LPI 6) determined by FQA from an average 8000 cells per sample. Each histogram bar represents the average proportion (%) of cells in a given length class (or bin) from two transgenic lines (three plants each) and six wild-type plants. Numbers in brackets are the number of fibres counted for selected bins. **(D) **Average fibre length determinations at three stages of development (LPI 6, LPI 21, LPI 43). Bars indicate average length ± SD of 6 samples analyzed for each treatment. * indicate fiber counts (D, whole population or C, bins) that differed significantly between transgenic and control trees (one-way ANOVA at *p *< 0.05).

The vascular organisation of petioles from mature leaves was examined to further investigate the altered development. Cell wall autofluorescence associated with lignin accumulation was observed in transverse sections under UV-illumination, and clearly indicated that the distribution of fibres and vessels was altered in the transgenic trees (Figure 4A-B). The ratio of fibres (small lignified cells; Figure 4) to vascular elements (large lignified cells) was 0.80 in the transgenic trees, compared to 1.55 in the controls (*p *< 0.001).

Furthermore, quantitative determinations of fibre lengths in stems and petioles through FQA (Fiber Quantitative Analyser, Methods) showed that petiole fibres from immature (LPI 6) and mature leaves (LPI 21 and LPI 43) were slightly, but significantly shorter in the transgenic trees (*p *< 0.05, Figure 4D). Shorter fibre classes < 0.5 mm were over-represented in the transgenic trees, whereas longer fibre classes (from 0.75 to 1, from 2.0 mm and up) were significantly under-represented compared to the control trees (*p *< 0.05, Figure 4C). The fibre length classes from primary stems (internode between LPI 5 and LPI 6) followed a similar distribution pattern, but the mean fibre length was not significantly different between controls and transgenics. Secondary xylem fibres from the main stem, which were sampled from the same internodes as the petioles (LPI 6, LPI 21, LPI 43), did not differ between the transgenic and wild-type trees.

### Effect of *PtaHB1 *over-expression on the transcriptome

Primary stem tissues from two control lines (two trees per line, n = 4 individual samples) and two *PtaHB1 *transgenic lines (two trees per line, n = 4 individual samples) were compared using a 3.4 K low redundancy cDNA microarray (GSE24703 for raw data on GEO database). A total of 48 transcripts that accumulated differentially were expressed with a false discovery rate (FDR, [30]) threshold set to 5% (*q *< 5.00) (SAM package release 3.0; [31]). Out of the 48 significantly misregulated genes, 8 transcripts were up-regulated and 40 were down-regulated in the transgenic trees (Table 1). The portion of the stem that we targeted in this analysis is also the part of the tree where petioles are actively developing and growing. Approximately one-third of the misregulated genes (14 out 48) had strong statistical support (*q *< 0.001). However, the fold-change of all genes identified was less than two (-1 < M < 1).

RT-qPCR analyses were carried out with gene-specific DNA primer pairs representing 20 putatively misregulated genes, to confirm the microarray data. These analyses used the same RNA samples as those used for microarray profiling plus two additional biological replicates (n = 6). Fold difference ratios from RT-qPCR results showed that twelve transcripts were congruent with the microarray results, while four genes gave no difference and four genes yielded conflicting results (Table 2). Data indicate that we were able to validate a subset of these misregulated sequences by RT-qPCR, which is consistent with a previous study reporting low rates of RT-qPCR validation when microarray fold-changes are less than two [32]. Thus, our relatively low validation rate is not surprising and could be explained by other factors, including cross-hybridisation of closely related genes to the cDNA probes, for which we could not account [33].

**Table 2:** Misregulated gene profiles from microarray analysis comparing transgenic UBI::PtaHB1 and wild-type lines and RT-qPCR validations.

**Poplar Gene ID**.		Functional Annotation		Microarray results		RT-qPCR validation	
**EST** **(Genbank)**	**Populus trichocarpa Genome V 2.0^1^**	**E value**	*POPTR BlastN **NCBI BlastX	**q-value (%)**	**M (log2 fold difference)**	**M (log2 fold difference)^2^**	**SD**

CN517570	POPTR_0004s23850	E-45	*predicted protein	3.065	-0.261	N/A	

CN517617	POPTR_0006s12510	0	**s-adenosylmethionine synthase 6	2.384	0.333	-0.833	0.453

CN517648	POPTR_0005s11070	E-120	**Peroxidase (PO3)	0.000	-0.275	0.017	0.195

CN517711	POPTR_0004s24390	E-90	*predicted protein	3.065	-0.213	N/A	

CN517879	POPTR_0001s28710	E-42	**Serine/threonine protein kinase	0.000	**-0.488**	-0.090 *	0.693

CN518033	POPTR_0010s14250	0	*predicted protein	3.065	-0.364	N/A	

CN518196	scaffold_6:22787285..22789257	E-05	**protein kinase	2.258	-0.179	N/A	

CN518487	POPTR_0010s12680	E-91	**mitochondrial beta subunit of F1 ATP synthase (PtrAtpB)	2.384	0.320	N/A	

CN518490	POPTR_0004s22020	E-53	**Fasciclin-like AGP 10	3.065	**-0.545**	-0.678 *	0.365

CN518917	POPTR_0010s05180	E-27	**putative polygalacturonase, pectidase	2.600	-0.200	N/A	

CN518924	POPTR_0015s00430	E-41	**Plastid-specific 30 S ribosomal protein 1	3.575	-0.293	N/A	

CN518966	POPTR_0003s13440	E-73	**progesterone 5-beta-reductase-A	2.384	0.323	N/A	

CN519065	POPTR_0008s14360	E-171	*Myoinositol oxygenase, Aldehyde reductase	0.000	**0.449**	0.323 *	0.838

CN519230	POPTR_0015s03670	0	*predicted protein	2.258	**-0.272**	-0.044 *	0.837

CN519263	POPTR_0022s00750	E-32	**Lactoylglutathione lyase, Glyoxalase putative	3.651	-0.204	N/A	

CN519295	POPTR_0012s14780	E-30	**Coatomer delta subunit	3.065	-0.266	N/A	

CN519368	POPTR_0004s21650	0	*phosphate-responsive 1 family protein	0.000	0.567	-0.246	0.404

CN519565	POPTR_0001s22700	E-55	**leucine rich protein, Brassinosteroid insensitve 1-associated receptor kinase (BAK-1)	2.768	**-0.314**	-0.710 *	0.326

CN520095	POPTR_0005s22210	E-108	**Oxidoreductase activity protein	0.000	0.390	-0.803	0.446

CN520368	POPTR_0008s06940	E-41	**Cys-3-His zinc finger protein	4.086	**-0.332**	-0.044 *	0.619

CN520805	POPTR_0009s02400	E-93	*leucine rich protein **Brassinosteroid insensitve 1-associated kinase repector, (BAK-1)	0.000	**-0.203**	-0.424 *	0.344

CN521180	POPTR_0001s13540	E-50	**Lactoylglutathione lyase/glyoxalase 1 family protein	0.000	**-0.281**	-0.287 *	0.432

CN521321	POPTR_0007s10200	E-14	**hydrolase, alpha/beta fold family protein	2.200	-0.227	N/A	

CN521367	POPTR_0006s29050	E-27	**ABC1 family protein	3.065	-0.255	N/A	

CN521610	POPTR_0009s12310	E-86	*Predicted protein	4.086	-0.520	0.059	0.591

CN521704	POPTR_0010s24930	E-21	**DnaJ homolog	3.651	-0.233	N/A	

CN521866	POPTR_0011s04190	E-49	**Armadillo/beta-catenin repeat family protein	0.000	-0.243	N/A	

CN522073	POPTR_0017s06630	E-29	**EXS family protein	0.000	-0.259	N/A	

CN522222	POPTR_0009s10300	E-142	*C3HC4 ring Zn-finger, Anaphase-promoting complex (APC), subunit 11	3.065	-0.258	N/A	

CN522424	POPTR_0008s23260	E-109	*Ethylene response factor (ERF35) Pt-RAP2.4	2.768	-0.358	N/A	

CN522566	POPTR_0005s12510	E-163	*Unknown function	0.000	-0.407	N/A	

CN522696	POPTR_0012s09650	E-81	**4-coumarate-coa ligase (Ptr4CL9)	2.768	-0.214	N/A	

CN522933	POPTR_0005s19400	E-21	**Branched-chain amino acid aminotransferase, putative	0.000	-0.209	N/A	

CN522970	POPTR_0005s27930	E-14	**bZIP transcription factor family protein	3.651	**-0.297**	-0.237 *	0.379

CN523006	POPTR_0006s19770	E-48	**phytocyanin-like arabinogalactan-protein	0.000	0.613	-0.890	0.762

CN523531	POPTR_0006s23940	E-104	**phytanoyl-CoA hydroxylase (PhyH) glycoproteins AGP19	3.065	**-0.299**	-0.330 *	0.484

CN521717	POPTR_0002s14730	E-26	**Transketolase	0.000	0.261	N/A	

CN523609	POPTR_0011s13810	E-126	*Translation initiation factor activity SUI1	3.065	**-0.281**	-0.192 *	0.560

CN522357	POPTR_0007s08390	E-136	**Elongation factor EF-2	3.065	**-0.336**	-0.533 *	0.427

HO702741	POPTR_0007s12770	0	*Unknown function	0.000	**-0.397**	-0.679 *	0.300

HO702768	POPTR_0009s13750	E-28	**Farnesylated protein	2.145	-0.388	N/A	

HO702822	POPTR_0010s00900	E-37	**AP2/Ethylene response factor domain-containing transcription facctor	3.651	-0.517	-1.034	0.860

HO702830	POPTR_0010s01590	E-160	*Late embryogenesis abundant protein 3	3.065	**-0.868**	-0.983 *	0.561

HO702837	POPTR_0015s06030	E-117	*Unknown fonction	3.065	-0.234	N/A	

HO702874	POPTR_0010s11840	E-66	*DUF26	2.600	-0.240	N/A	

HO702885	POPTR_0002s20260	E-61	**Ethylene receptor 1 (ETR1)	3.651	-0.280	N/A	

HO702895	scaffold_20:856315..856454	E-45	*Unknown function	0.000	-0.360	N/A	

HO703041	POPTR_0008s20950	E-128	*DUF588, Nitrate, Iron, Fromate dehydrogenase, integral membrane protein	3.065	-0.914	N/A	

^1 ^Sequence identified from poplar genome V1.1; was not found in V2.0

^2 ^Microarray results validated by RT-qPCR are highlighted in black, while grey highlights indicate results were not validated by RT-qPCR. N/A not assayed.

Microarray results are for primary stem tissues. Fold differences (M) were derived from two independent transgenic lines (two plants per line) (see methods, raw data available on NCBI GEO database, accession # GSE24703).

The predicted functions of the misregulated transcripts in the *PtaHB1 *transgenics were examined and separated into four categories: growth factor-related, cell wall-related, membrane trafficking, and general functions. The growth factor group included sequences related to brassinosteroid action, which are putative leucine-rich BAK1-like proteins (CN520805, CN519565). These genes were down-regulated and suspected to be involved in steroid signal transduction [34]. Genes for ethylene perception and response were also down-regulated (HO702822, CN522424, and HO702885). Cell wall-related sequences were an abundant category of down-regulated transcripts. Other sequences related to cell expansion and cell proliferation were down-regulated, including sequences encoding two fasciclin-like proteins (CN518490) [35] two glyoxalases (CN519263, CN521180) [36], a farnesylated protein (HO702768) [37] and an elongation factor 2 (CN524724). The down-regulated sequences also included a 4CL gene (CN522696) that is involved in the synthesis of G-lignin precursors, and which is consistent with the decrease in auto-fluorescent fibres [38]. The up-regulated sequences encoded transketolase-like proteins putatively involved in isoprenoid biosynthesis (CN523609) [39] and in decreasing cell proliferation in preparation to dormancy [40].

The impact of *PtaHB1 *and *PtaHB7 *transgene expression on the other HD-Zip III gene transcripts was investigated in the transgenic poplars (Table 1). In general, the *UBI::PtaHB1 *constructs led to decreased transcript accumulation of all other HD-Zip III genes. However, the number of RNA molecules was quite variable and the effect was significant only for *PtaHB5 *(Student's t test, mean log_2 _ratio -0.7920, p = 0.0430). In *UBI::PtaHB7 *transgenic trees, the HD-Zip III genes had slightly increased transcripts levels, but only *PtaHB1*, *PtaHB3 *and *PtaHB5 *were significantly upregulated (mean log_2 _ratio 0.5521, p = 0.0318; 0.7675, p = 0.0148 and 0.7263, p = 0.0046).

### Accumulation of some, but not all HD-Zip III transcripts is linked to auxin in Poplar

Given that some HD-Zip III genes have been linked to auxin transport during vascular development [12] and that *PtaHB1 *overexpression affected the accumulation of several transcripts related to growth regulators, we examined whether or not auxin influenced transcript accumulation of HD-Zip III genes in developing secondary xylem of young poplar trees. Removal of the stem apex, which is the primary source of auxin in the plant, significantly decreased the transcript level of *PtdHB5 *in the xylem tissue by nearly four-fold (mean log_2 _ratio = -1.9739) and had a similar effect *PtdHB7 *but it was not found to be statistically significant (mean log_2 _ratio = -1.6421; p-value = 0.0776), and did not affect *PtdHB1 *(Table 3). The application of N-(1-naphthyl) phthalamic acid (NPA) to a portion of the stem undergoing secondary growth (see Methods) significantly decreased transcript abundance for *PtdHB1*, *PtdHB5 *and *PtdHB8 *(mean log_2 _ratios of -1.2010, -2.0375, -0.7031, respectively). The only gene affected by both treatments was *PtdHB5*.

**Table 3:** Differential transcript level of HD-Zip III genes in developing secondary xylem from P. trichocarpa × P. deltoides (clone H-1111) following removal of the apex or application of an auxin transport inhibitor (NPA), compared to untreated controls.

Gene	Treatment	Log_2_-fold difference	SD^1^	*p*-value^2^
*PtdHB1*	apex (-)	-0.5062	1.17	0.1092
	NPA	-1.201	1.09	0.0270*

*PtdHB2*	apex (-)	0.4671	1.17	0.4777
	NPA	-0.4111	0.89	0.4307

*PtdHB3*	apex (-)	0.1637	0.91	0.4663
	NPA	-0.4473	0.71	0.4762

*PtdHB5*	apex (-)	-1.9739	0.69	0.0461*
	NPA	-2.0375	1.22	0.0496*

*PtdHB6*	apex (-)	-0.9145	0.91	0.1346
	NPA	-1.4915	1.14	0.1090

*PtdHB7*	apex (-)	-1.6421	0.76	0.0776
	NPA	-1.3204	1.26	0.1725

*PtdHB8*	apex (-)	-0.5686	0.98	0.1488
	NPA	-0.7031	1.93	0.0455*

^1^SD is one standard deviation.

^2^p-value is based on Student's *t *test, N = 2 pools of 3 samples); * indicates the treatment had significant effect a threshold of 0.05

## Discussion

Vascular development is a finely tuned process that is integral to primary growth, i.e., stem elongation, as well as secondary growth, i.e., radial or diameter growth. The differentiation and growth of the primary vasculature derives from the apical meristem, whereas secondary vascular tissues derive from the cambium. The specific spatio-temporal control and action of regulators enable the coordinated differentiation of the vasculature and other tissues during plant development. In plant model systems such as *Arabidopsis *and *Zinnia*, it has been established that key events underlying vascular differentiation involve a few different HD-Zip III transcription factors. This small family of regulators are known for their overlapping expression profiles and their functional redundancy. The aim of this study was to develop insights into the role of HD-Zip III genes in secondary xylem formation in forest trees. We examined the HD-Zip III gene family in two unrelated tree species belonging to the angiosperms (*Populus *spp.) and the gymnosperms (*P. glauca*).

### Distinct HD-Zip III gene family evolution in gymnosperm and angiosperm trees

Gene sequences isolated from the moss *P. patens *with features typical of HD-Zip genes of class I, II, and III clearly indicate that they were acquired early in plant evolution [41]. The sequence analyses presented here (Figure 1) are consistent with the idea that HD-Zips have evolved through gene or genome duplications and potential gene losses [21,22].

The phylogenetic tree we described (Figure 1) included four full length HD-Zip III cDNA sequences of *P. glauca *and was similar but not entirely congruent with the tree topology previously predicted with a Bayesian procedure that used full length and partial cDNA sequences from *P. taeda *[21,22]. On one hand, previous authors have reported that gymnosperm HD-Zip III sequences could be assigned to both the C8 and RVB clades defined in angiosperm plants. On the other hand, they showed that gymnosperms also formed two independent clades not represented in angiosperms. Our results are consistent with the existence of gymnosperm clades with representatives from *Pinus *and *Picea*. These findings support the hypothesis that modern HD-Zip III family structure derives from four ancestral sequences, and that two of the ancestral sequences have been lost in angiosperms leading to clades C8 and RVB, whereas all four clades have potentially been retained in gymnosperms. However, our finding that the RVB clade lacked conifer sequences and the lack of a reference gymnosperm genome sequence led us to conclude that further analyses are needed to confirm whether or not gymnosperms are in fact represented in the RVB subclade.

Poplars have three more HD-Zip III sequences than Arabidopsis, which is consistent with the inferred genome evolution of the former [26]. The poplar sequences clearly formed four pairs of closely related paralogues. The salicoid plant lineage that gave rise to the family *Salicaceae *(including *Populus *spp.) appears to have undergone a relatively recent genome-wide duplication and reorganisation [26], whereas Arabidopsis is thought to have undergone genome size reduction [42]. These different evolutionary paths could have led to the loss of certain functions as well as neofunctionalisation or subfunctionalisation within the angiosperms.

### Transcription profiles identify HD-Zip III putatively involved in vascular development

Delineating the potential role of HD-Zip III genes in regard with vascular development is aided by comparing RNA transcript accumulation in different organs, tissues and cell types, despite the overlapping profiles that may be observed within the family. Members of the C8 clade have been most strongly linked to vascular development and have not been implicated in leaf formation as such. In Arabidopsis, *AtHB-15/CNA *is expressed in procambial cells where it is involved in early initiation of vascular cells, and has been implicated in embryo polarity. The *AtHB-8 *gene product has been shown to promote the proliferation and differentiation of xylem cells. Its expression also localizes to pro-cambium cells, in addition to being modulated by auxin [16]. The transcripts corresponding to the three *P. glauca *sequences we assigned to the C8 clade were detected in all tissues but preferentially in differentiating secondary vascular tissues both in the stem and in the roots. This observation may represent evidence in support of the phylogenetic position of *Picea *sequences *PgHB-3 *to *PgHB-5*, along with several other gymnosperm sequences, in clade C8 rather than RVB. In *Populus*, there are four C8 sequences *PtdHB5 *to *PtdHB8 *with varied transcript accumulation profiles in vascular tissues. The accumulation of *PtdHB5 *and *PtdHB-6 *transcripts were also clearly preferential to secondary xylem tissues. In contrast, the paralogous sequences *PtdHB7 *and *PtdHB8 *have very dissimilar profiles and were distinctly not preferential to secondary xylem. These transcript accumulation profiles of *PtdHB7 *and *PtdHB8 *indicated that *Populus *C8 sequences may have undergone relatively recent neofunctionnalisation or subfonctionnalisation, compared to the pair of *PtdHB5 *and *PtdHB6 *which share the most similar expression patterns. Overall, it appears that gene duplications found in gymnosperm C8 clade, and even the more ancient duplications at the family level (*PgHB6*), have not led to strong diversification of expression profiles compared to that observed in angiosperms.

Lateral organ formation has been assigned to RVB clade that includes *REV*, *AtHB-9 *(*PHB*) and *AtHB-14 *(*PHV*). The closely related genes *PHB *and *PHV *are involved in leaf polarity, while *REV *has been implicated in several developmental processes, including vascular cambium identity and activity, as well as fibre differentiation. Two putative homologues of *Arabidopsis REV *genes have been detected in the genomes of *Populus*, *Z. elegans, O. sativa *and *Z. mays *L. [43,44]. The functions of the *Zinnia REV *homologues appear to have diverged, with one being implicated in vascular development and the other in lateral organ formation [9]. In contrast, the *Populus HB1 *and *HB2 *have similar transcript patterns, except that *HB2 *transcripts accumulate more strongly in maturing leaves (Figure 2B). Arabidopsis may represent a unique case with a *REV *paralog potentially having been lost during ancestral genomic rearrangements [42], and resulting in a gain of function for the remaining *REV *sequence in developing xylem and leaves. Ko *et al*. (2006) [6] found that *PtdHB1 *was associated with secondary growth in poplar stems and hypothesised that HD-Zip III genes played a role in secondary xylem differentiation in trees. Our expression survey indicated that *PtdHB1 *transcripts are present at a similar level in the apex, primary stems, secondary xylem, and young roots.

### Poplar HD-zip III genes play a role in fibre development

Constitutive over-expression of the poplar *PtaHB1 *gene in poplar led to greater transcript abundance corresponding to this gene, and resulted in shorter petioles and a wider angle between the stem and adaxial side of the petiole. The fibres with reduced lignification and shorter length suggested that development of primary xylem fibres was either impaired or delayed in the transgenic trees. Our hypothesis is that the increased angle between the petioles and the stem is caused by a delayed or incomplete fibre development relative to leaf expansion. Asynchronous development may cause the petioles to lack the necessary strength to support a fully expanded leaf. This phenotype bears a resemblance to that of the *ifl-1 *mutant (*REV *gene) in which interfascicular fibre development is impaired and inflorescence stems lack sturdiness [43]. Moreover, it has been observed that *REV *gain-of-function promotes xylem differentiation and accumulation, leading to dysfunctional vascular patterning [13]. It thus appears that the *PtaHB1 *over-expression phenotype is closer to the *ifl-1 *mutant phenotype than the *REV *gain-of-function phenotype. This result contrasts with our initial hypothesis that *PtaHB1 *over-expression might promote fibre differentiation.

Furthermore, the vascular phenotype observed in *PtaHB1 *transgenic poplars was not consistent with previous observations that over-expression of non-mutated HD-Zip III genes had no effect on plant development because of gene silencing by microRNAs. In *Arabidopsis*, all of the HD-Zip III family transcripts are targeted and negatively regulated by microRNAs (MiR165/166) found in multiple copies in the genome [6]. Moreover, the over-expression of the HD-Zip III sequences was shown to trigger the production of these microRNAs [45]. An hypothesis to explain our observations may be that HD-Zip III *PtaHB1 *over-expression triggered the accumulation of microRNAs that down-regulated the other members of the HD-Zip III family. Transcript accumulation of the other poplar HD-Zip III sequences provided evidence in support of this hypothesis. Interestingly, *PtaHB7 *over-expression did not appear to have a similar effect on other family members.

It has long been known that fibre differentiation or stem elongation is controlled by growth factors such as auxin, GA and brassinosteroids, and that overproduction of auxin decreases primary stem elongation in *Arabidopsis *[46]. The *REV/IFL-1 *gene of *Arabidopsis *has been implicated in polar transport of auxin [43]. Considering the known relationship between plant growth regulators and HD-Zip III genes, the delay in petiole elongation in *PtaHB1 *transgenics may be linked to a perturbation of growth regulation activity. For example, auxin accumulation may be shifted due to altered polar transport. Our observations in young wild-type poplar trees suggested a putative link between auxin and the expression of three different HD-Zip III genes (*PtdHB1*, *PtdHB5 *and *PtdHB8*), the transcript levels of which were affected by the application of an auxin transport inhibitor (Table 3). This observation is consistent with the fact that those gene transcripts are well represented in secondary xylem (Figure 2B). Plant decapitation (from the apex to LPI 3 inclusively) significantly down-regulated transcripts of *PtdHB5 *and had a smaller effect on *PtdHB7 *transcripts. These results are partially consistent with previous reports from *Arabidopsis*, where *AtHB-8 *was clearly modulated by auxin. Gene regulation may have evolved differently in poplar compared to Arabidopsis, reflecting the developmental and physiological differences between a woody perennial plant and an herbaceous annual.

The differentially expressed sequences identified in the *PtaHB1 *over-expressing poplars were classified into a few broad categories based upon putative functional assignments. Most of the transcripts were down-regulated and included sequences related to development pathways: brassinosteroid and ethylene growth regulators, ethylene perception and response, and putative steroid signal transduction proteins, in addition to cell wall-related and cell expansion or cell proliferation proteins. Many of the sequences have putative functions that appear consistent with developmental activities of *IFL1/REV *in *Arabidopsis*. They also appear to be consistent with the functions or pathways related to the phenotype observed in transgenic poplars.

## Conclusions

The analysis of HD-Zip III genes in vascular development has been complicated by the complex interactions between family members and the pleiotropic nature of the mutant phenotypes [22]. Nevertheless, the experiments and findings reported here contribute to confirming the involvement of this group of genes in woody plants, including primary and secondary vascular development. Taken together, the observations indicate that HD-Zip III gene family structure has considerably diverged between angiosperms and gymnosperms, and suggest that individual genes within each taxonomic group have acquired distinct and specialised functions, some of which are related to secondary xylem growth. The phenotype observed upon over-expression of a wild-type poplar *PtaHB1 *gene represents a departure from previous reports in *Arabidopsis*, where gene silencing by miRNAs suppressed potential effects of over-expression. The transgenic poplars likely represent a useful system to continue investigating the functions of *PtaHB1*. Transcript profiling identified a set of sequences which may be targets of *PtaHB1 *or perhaps lay downstream of *PtaHB1 *in the developmental cascade. Microarray profiling experiments using whole-genome arrays and targeting of other plant tissues related to the transgenic phenotypes would likely help to consolidate the list of candidate targets.

Future studies could continue to explore and compare more broadly the role of HD-Zip III genes in primary and secondary vascular growth of woody plants, particularly the poplar *PopHB5 *(closest homologues to CNA) which appeared the most specific to secondary xylem of all other poplar HD-Zip III. In this regard, HD-Zip III knock-downs (RNAi or anti-sense) could potentially help to clarify the putative role of poplar HD-Zip III genes. Genes encoding regulators of wood formation potentially represent a rich source for identifying genetic markers of wood properties that could used in targeted breeding and selection. It typically takes 10-15 years or more to grow trees to the point where they can be selected to develop improved or new varieties; therefore, if such markers are proven effective, they could potentially reduce the selection time and cost.

## Methods

### Plant material and growth conditions

*Picea glauca *(Moench) Voss (white spruce) tissues were obtained from two sources. The gene isolation work used plantlets of clone Pg-653, which are produced by somatic propagation by the Canadian Forestry Service (Klimaszewska *et al.*, 2001). For the transcript-level survey, samples were obtained from two wild field grown 33-year-old trees in a progeny trial that had been established near Québec City. Two hybrid poplar clones were used: *Populus trichocarpa *(Torr. & Gray) × *P. deltoides *(clone H11-11, for gene expression experiments) and *Populus tremula *L. × *P. alba *L. clone INRA clone 717 1-B4 (referred to as clone 717 for transgenic analysis). Rooted softwood poplar cuttings were produced in 25 cm^3 ^pots protected in clear plastic bags, transferred to 3 L pots after five weeks, and maintained in a greenhouse. Both the spruce and poplar plants were grown in a greenhouse with 16 hours light per day, with a temperature regime of 22/17°C (day/night), and relative humidity of at least 70%. Natural daylight was supplemented with light from HQI-TS 400W/DH metal halogen lamps (Osram, Munich, Germany). Plants were fertilised weekly with 1 g L^-1 ^20/20/20 (N-P-K) and supplemented with calcium every two weeks.

### Tissues for transcript profile survey

All tissues were frozen in liquid N_2 _immediately upon removal from the tree and stored at -80°C until further use. Several organs/tissues were isolated from two 33-year-old field-grown white spruce *P. glauca *for transcript accumulation profiling. These included the terminal leader (1T), young needles from the upper crown (YN), differentiated secondary phloem (2P) and xylem (2X), as well as bark (B) tissues collected from three 30-40 cm bolts taken from the lower third of the main stem. The secondary phloem and xylem issues were scraped with scalpel immediately after removing the bark: 2P was scrapped from the exposed inner side of the bark and the 2X from surface of the exposed wood. This method relies on cleavage of tissues at the cambial zone and yields relatively pure tissue types, although the purity of 2X and 2P samples were not verified as such. Similarly, tissues from large roots, including differentiating xylem (R2X) and phloem (RPP; with phelloderm) were collected taken in a one-meter radius from the base of the stem. Each sample was kept separate for total RNA extraction. Tissues were also collected from two trees of clone H11-11 selected to be similar in size, i.e., average of 80 cm tall and possessing at least 25 leaves with a plastochron index (LPI) greater than zero [47]. LPI 0 denotes a leaf blade that is 1 cm long and is undergoing laminar expansion. The tissues consisted of: Apex LPI 0 without leaves (A); shoot tips up to and including LPI 1 (1T); young leaves up to LPI 3 (YF); mature leaves LPI 15-16 (ML); old leaves LPI 30-31 (OL), and differentiating xylem and phloem scrapped with a scalpel from the portion of the main stems exhibiting secondary growth (2X and 2P, respectively) between LPI 15 and LPI 20; and actively growing white coloured roots (R).

### Auxin-related treatments in poplar

Two treatments were applied to young poplar trees (*P. trichocarpa *× *P. deltoides *H11-11, described above) averaging 80 cm in height in order to assess the potential effect of auxin on HD-Zip III transcript accumulation in poplar. Six randomly selected trees which possessed at least 25 leaves with a plastochron index (LPI) greater than zero were assigned to each treatment and to the control untreated group. The first treatment removed the shoot apex, which involved cutting off the top part of plants down to LPI 3 inclusively. The second treatment applied an inhibitor of polar auxin transport, N-(1-naphthyl) phthalamic acid (NPA, 1 mM for treatment and 0 mM for control) mixed with lanolin as a carrier. The mixture of NPA and lanolin was placed on the bark (after removing the layer of cuticle), entirely covering a segment of the stem of 2.5 cm centered at the internode LP15, and covered with paraffin film. Secondary xylem tissues were collected 72 h after the treatments were applied, from internodes LPI14 to 16 and consisted of whole stem segments without the bark. For the NPA treated trees, the sampling consisted of stems segments without bark 3 cm above and 3 cm below the region of NPA application (LPI15 was excluded). The transcript accumulation data (see below) obtained from these tissues were analysed using Student's t test applied to each gene separately comparing the treated and untreated plants. For the NPA treated trees, tissues obtained below and above the region of application were analysed separately and the data were averaged for each tree.

### RNA extraction, sequence isolation and phylogenetic analyses

Total RNA extractions were carried out using the CTAB method of Chang *et al*. (1993) [48] from spruce and poplar tissues, which were stored at -80°C until used. The quantity and integrity of total RNA were evaluated by spectrophotometry (OD 260/280 ratio of 1.8:1 to 2.1:1), and by using a Bioanalyzer 2001 using an RNA 6000 Nano Kit (Agilent Technologies, Palo Alto, CA, USA) to achieve a 28S:18 S ratio of 1.2:1 to 2.2:1 and an RNA integrity number (RIN) above 7.

Partial HD-Zips III gene sequences were identified among EST (Expressed Sequenced Tags) obtained from the conifers *P. taeda *[49] and *P. glauca *[50] through BLAST analysis (Basic Local Alignment Search Tool). Completed coding sequences and untranslated 3' and 5' region (UTRs) were isolated by using PCR-cloning with degenerate primers or 3' RACE, 5' RACE (SMART RACE cDNA Amplification, BD Biosciences Clontech, Mountain View, CA, USA) or both cloning methods with mRNAs from needles or xylem. The cDNAs were cloned in pCR2.1 with the TA cloning kit (Invitrogen, Carlsbad, CA, USA), then sequenced with a 16-capillary genetic analyzer (ABI Prism 3130XL and an ABI Prism 3100XL, Applied Biosystems, Foster City, CA, USA). Public databases were searched by use of the BLAST algorithms to identify HD-Zip III sequences in the Poplar genome (JGI, *P*. *trichocarpa *v1.0, [26] and updated v2.0 http://www.phytozome.net/poplar), and the non-redundant NCBI database (nr; http://blast.ncbi.nlm.nih.gov/). Corresponding cDNAs were amplified from first-strand cDNA which had been derived from pooled total RNA extracted from developing secondary xylem, tips and young leaves (*P. trichocarpa *× *P. deltoides *and *P. tremula *× *P. alba*). The identity of each cDNA clone was confirmed by complete and partial sequence comparisons with the poplar genome and previously identified EST sequences [6].

The predicted HD-Zip III amino acid sequences from multiple species were aligned using *ClustalW *algorithm [51], which is included in the *Bioedit *software [52], and then refined manually. Phylogenetic analyses used the alignment of complete amino acid sequences via *MEGA *software [53]. A Neighbour-Joining phylogenetic tree [28] was constructed based on a Poisson correction model and pair-wise deletion algorithm (1,000 bootstrap replicates). The phylogenetic tree was rooted with three members of other HD-Zip class 1 and 4 sequences found in the *Genbank *database [54].

### RT-qPCR procedures

Total RNA (2 μg) was DNase-treated and reverse-transcribed using the SuperScriptII First-Strand Synthesis System for RT-PCR following manufacturer's recommendations (Invitrogen, Carlsbad, CA, USA). The resulting first-strand cDNA was diluted 1:10 in deionised water before quantitative PCR determinations. An aliquot of cDNA equivalent of 20 ng of total RNA was used per 20 μL of PCR reaction. Amplifications were performed in a QuantiTect™PCR SYBR^® ^Green Kit (Qiagen, Mississauga, ON, Canada) with 0.3 μM of 5' and 3' primers, with a DNA Engine Opticon TM 2 System (MJ Research Inc., Ramsey, MN, USA) following the manufacturer's instructions. Primers were designed with Primer3 software [55], with a melting temperature (Tm) between 55°C and 62°C, and produced amplicons between 100 and 250 bp (Additional file 1). The poplar RT-qPCR primers were tested on the clones H11-11 and INRA-717. Amplification reaction efficiencies were between 90 and 105% for each primer pair. The thermal-cycling parameters were as follows: 95°C for 15 min; 45 cycles of 94°C for 15 sec, 55°C for 1 min and 72°C for 40 sec; followed by a melting curve analysis from 54°C to 95°C with increments of 0.1°C per step to verify the specificity of the amplification and presence of primer dimers. The number of target copies in each sample was determined from Ct values (cycle threshold values) using a linearised plasmid or purified PCR product to produce a standard curve, which was obtained by averaging values from several runs. Two to six independent sample assays were performed and each sample was loaded in duplicate. Results were normalised relative to the absolute RNA used in a single reaction and with the transcript level of a reference gene. The spruce data were normalised to the reference of transcript level gene encoding *Elongation factor 1 alpha*, *EF1a*, (AJ132534) [56]. The *EF1a *gene were strongly expressed and showed low variation between spruce tissues [57]. In poplar, the reference was a *cell division cycle 2 *gene homologue, *CDC2 *(AF194820) [58]. *CDC2 *cycle threshold values (Ct) averaged 18.5 + 0.5 (Standard Deviation), which is the experimental error range of the RT-qPCR cycler device. For statistical analyses of the RT-qPCR data from auxin-related treatments and transgenic experiments (Tables 1, 2, 3) a log_2 _transformation was applied to the number of target copies.

### Agrobacterium transformation and growth of transgenic poplars

Over-expression constructs were obtained by inserting the complete coding sequences of *PtaHB1 *and *PtaHB7 *from *P. tremula *× *P. alba *clone INRA-717 between the maize ubiquitin promoter [59] and a 35 S terminator into the pCambia1305.2 vector http://www.cambia.org. The resulting plasmids were then transferred into *A. tumefaciens *strain C58 pGV2260 [60].

For transformation, *in vitro *plantlets of the hybrid poplar clone INRA-717 were micropropagated on hormone-free 1/2 MS medium [61] supplemented with vitamin D3. Internodes from *in vitro *plantlets were co-cultivated with the engineered *A. tumefaciens *according to the method of Leple *et al*. (1992) [62], with the following modifications. After co-cultivation, the explants were decontaminated of *A. tumefaciens *with cefotaxim and transferred onto M2 medium containing cefotaxim alone [62]. Transgenic calluses were selected on M3 medium supplemented with hygromycin (10 mg L^-1^) and emerging shoots were transferred for rooting to 1/2 MS medium without hygromycin, screened for positive X-glucuronidase activity [62], and assayed for poplar *PtaHB1 *and *PtaHB7 *mRNA accumulation by RT-qPCR. Shoots with elevated *PtaHB1 *and *PtaHB7 *mRNA levels relative to WT control were transferred to the greenhouse where they were grown under a photoperiod of 16 h of light, at 24/20°C (day/night). For each construct, four to six trees for each of six lines (independent transformation events), and 10 to 15 WT INRA-717 trees were propagated and carried forward together for phenotypic determinations.

### Phenotype characterisations of transgenic poplars

Morphometric measurements were performed on two trees from three independent transformation events per line and six control trees (WT). Internode length, petiole length, and petiole angle were measured from LPI 1 to the bottom of each tree. Vascular tissue organisation was visualised on samples taken from fresh stem and petiole sections from LPI 20. Samples were fixed in 4% paraformaldehyde - 2% glutaraldehyde cacodylate buffer (pH 7.2) for 12 hours under vacuum, and stored at 4°C before embedding in paraffin blocks. Microtome cross-sections (10µm thick) were mounted on glass slides; after the paraffin was removed, the sections were rehydrated for observation under UV light on an Olympus BX51 microscope (Olympus, Montreal, QC, Canada). Fibre and vessel distributions in petioles were visualised by lignin autofluorescence and counted (75 to 150 cells per field imaged). The average fibre to vessel ratio was computed from four transgenic plants and four WT plants. Fibre Quality Analysis (FQA; OpTest Equipment, Hawkesbury, ON, Canada) used freshly debarked internodes, and intact petiole sections from LPI 6, LPI 21 and LPI 43. The samples were macerated in Franklin's solution until they were completely bleached and the fibres could be separated [63]. The FQA weighted, fibre length (lw) data (8000 individual fibres per petiole or internode) were obtained from three trees per transgenic line on six lines and on six control trees. For analyses, the data were placed into bins of 0.10 to 0.25 mm.

### Statistical analyses of phenotypic and RT-qPCR data

Statistical treatment of phenotypic data compared transgenic poplars transformed empty vector (control lines) and those transformed with the *PtaHB1 *gene construct. Except where otherwise noted, the data were not transformed and each tree was considered an individual experiment unit. Normality was confirmed and Student's *t *tests were used for petiole length and angle data (Figure 3), with each internode tested separately in SYSTAT 13 (Cranes Software International Ltd., Chicago, IL). Student's *t *tests were also used to compare the proportion fibres to vessels within the petioles, determined by using UV microscopy (Figure 4A and 4B). The fibre length data (FQA) were analysed by comparing the proportion (%) of fibres in each length class (bin) (Figure 4C) with a one-way ANOVA, on each bin independently, and also by comparing the overall fibre length data (Figure 4D). Before analysis the normality of the data was confirmed, and ANOVAs were carried out using procGLM in SAS (Version 9.01, SAS Institute Inc., Cary, NC).

For RT-qPCR data comparing control and *PtaHB1 *transgenic lines (Table 1, 2) and comparing the impact auxin-related treatments (Table 3) normality and Student's *t *tests were applied to log_2 _transformed numbers of transcript targets in SYSTAT 13 (Cranes Software International Ltd., Chicago, IL).

### Microarray RNA profiling

A poplar 3.4 K cDNA microarray was prepared by the ARBOREA project and is described in Pitre et al. (2010) [64]. RNA transcript profiling was carried for each of the transgenic constructs, with four samples from two independent transformation events compared with four control samples from two independent transformation events (empty vector controls). Primary stem tissues (LPI 0 to 5) were used for profiling *PtaHB1 *over-expressing lines and secondary stem xylem tissues (LPI 21 to 43) were used for the *PtaHB7*. The microarray hybridization methods were as described in Pitre et al. (2010) [64]. Each microarray hybridisation used 1 μg of total RNA that was amplified using the SuperScript™ Indirect RNA Amplification System (Invitrogen, Carlsbad, CA, USA) and 5 μg of the aRNA were labelled with Alexa Fluor^®^555 and 647 dyes (Invitrogen Carlsbad, CA, USA), for use in dye-swap experiments. The poplar 3.4 K microarray slides were pre-hybridised for 2 hours at 42°C in a solution containing 5× SSC, 0.1% SDS, 0.02% BSA (w/v), 0.01% herring sperm DNA (w/v), and 50% formamide. The slides were then washed twice in 0.1× SSC, once in water, rinsed in 2-propanol, and finally dried by centrifugation. The labelled targets (3.5 μL) were mixed with 52.5μL of hybridisation solution containing 5× SSC, 0.1% SDS, 0.01% herring sperm DNA (w/v), and 50% deionised formamide. The mixture was heat-denatured for 4 min at 95°C and cooled for 5 min on ice prior to hybridisation to the microarray. The microarray was then covered with a LifterSlip (Erie Scientific Company, Portsmouth, NH, USA) and placed in a hybridisation chamber II with increased depth (Corning, Lowell, MA, USA) and incubated for 12 h at 45°C in a model 1012 hybridisation oven (Shel Lab, Cornelius, OR, USA). After hybridisation, the slides were iteratively washed for 15 min in 2× SSC + 0.5% SDS, 0.5× SSC + 0.1% SDS and 0.1× SSC solutions at 45°C.

The slides were scanned using a ScanArray™Express scanner (Packard BioScience, Meriden, CT, USA) and the image files were analysed using QuantArray^® ^software (Packard BioScience, Meriden, CT, USA). Scan intensities were comparable between sets of slides for a given hybridisation. Data analysis was carried out using Bioconductor packages http://www.bioconductor.org distributed in R (R Development Core Team 2008). Median foreground intensity minus median background intensity was the response variable used for the statistical analysis. Data quality was assessed using graphical analysis tools in the *marray *and *olin *packages available in Bioconductor, and by assessment of within- and between-slide Pearson product-moment correlation coefficients (*r*), which were calculated both from the raw intensities and after normalisation. The composite normalisation method [65] was applied by using the two functions *maNorm2 D *and *maNormLoess *in the *marray *package [66]. We identified differentially expressed sequences with the SAM package release 3.0 [31] using a false discovery rate (FDR) [30] threshold set to 5% (*q *< 5.00). Data reported in the article are log_2_-ratios of Alexa Fluor^®^555/647, which are denoted M. Raw data are available in the Gene Expression Omnibus (GEO) database (accession number: GSE24703, http://www.ncbi.nlm.nih.gov/geo/).

## List of abbreviations

EST: expressed sequence tag; HD-ZIP: homeodomain leucine-zipper; RT-qPCR: reverse-transcription quantitative PCR;

## Authors' contributions

CLC drafted the manuscript, carried out the poplar sequence analyses, the phylogenetic tree construction, RT-qPCR analyses in poplar, transgenic poplar characterisations and microarrays results interpretations. FM carried out the spruce sequence analyses and RT-qPCR analyses in spruce. VR conducted the microarray RNA-profiling experiment. MO isolated PtrHB1 and PtrHB5 cDNA sequences. CL carried out plant tissue transformations and produced the transgenic trees. M-JM prepared vector plant tissue transformation. JEKC oversaw microarray development, participated in the manuscript revision. AS oversaw plant tissue transformations and production of transgenic trees, participated in the manuscript revision. JJM oversaw overall project and manuscript preparation. All authors read and approved the final manuscript.

## Supplementary Material

Additional file 1 **Gene predicted names, accession numbers and primers for RT-qPCR used in poplar and white spruce**. First column contains GenBank accession number, second column contains name of the genes cloned in poplar (POPTR_ID V2 of Populus trichocarpa) and white spruce, third column contains RT-qPCR primers forward and reverse. Click here for file

## References

[B1] Fukuda H Xylogenesis: Initiation, progression, and cell death Annual Review of Plant Physiology and Plant Molecular Biology 1996 47 299 325 10.1146/annurev.arplant.47.1.299 15012291

[B2] Telewski FW Aloni R Sauter JJ Stettler RF, Bradshaw HD, Heilan PE, Hinckley TM Physiology of secondary tissues of *Populus* Biology of Populus and its implications for management and conservation 1996 Ottawa, ON, Canada: NRC Research Press 301 329

[B3] Zhang SY Effect of growth-rate on wood specific-gravity and selected mechanical-properties in individual-species from distinct wood categories Wood Science and Technology 1995 29 451 465 10.1007/BF00194204

[B4] Izawa T Takahashi Y Yano M Comparative biology comes into bloom: genomic and genetic comparison of flowering pathways in rice and Arabidopsis Current Opinion in Plant Biology 2003 6 113 120 10.1016/S1369-5266(03)00014-1 12667866

[B5] Nieminen KM Kauppinen L Helariutta Y A weed for wood? Arabidopsis as a genetic model for xylem development Plant Physiology 2004 135 653 659 10.1104/pp.104.040212 15208411 PMC514101

[B6] Ko JH Prassinos C Han KH Developmental and seasonal expression of PtaHB1, a *Populus *gene encoding a class III HD-Zip protein, is closely associated with secondary growth and inversely correlated with the level of microRNA (miR166) New Phytologist 2006 169 469 478 10.1111/j.1469-8137.2005.01623.x 16411950

[B7] Aso K Kato M Banks JA Hasebe M Characterization of homeodomain-leucine zipper genes in the fern *Ceratopteris richardii *and the evolution of the homeodomain-leucine zipper gene family in vascular plants Molecular Biology and Evolution 1999 16 544 552 10331279 10.1093/oxfordjournals.molbev.a026135

[B8] Johannesson H Wang Y Engstrom P DNA-binding and dimerization preferences of *Arabidopsis *homeodomain-leucine zipper transcription factors in vitro Plant Molecular Biology 2001 45 63 73 10.1023/A:1006423324025 11247607

[B9] Ohashi-Ito K Kubo M Demura T Fukuda H Class III homeodomain leucine-zipper proteins regulate xylem cell differentiation Plant and Cell Physiology 2005 46 1646 1656 10.1093/pcp/pci180 16081527

[B10] Mukherjee K and TR Burglin MEKHLA, a novel domain with similarity to PAS domains, is fused to plant homeodomain-leucine zipper III proteins Plant Physiology 2006 140 1142 1150 10.1104/pp.105.073833 16607028 PMC1435804

[B11] Chandler JW Cole M Flier A Grewe B Werr W The AP2 transcription factors DORNROSCHEN and DORNROSCHEN-LIKE redundantly control *Arabidopsis *embryo patterning via interaction with PHAVOLUTA Development 2007 134 1653 1662 10.1242/dev.001016 17376809

[B12] Zhong RQ Ye ZH Alteration of auxin polar transport in the *Arabidopsis **ifl1 *mutants Plant Physiology 2001 126 549 563 10.1104/pp.126.2.549 11402186 PMC111148

[B13] Zhong RQ Ye ZH Amphivasal vascular bundle 1, a gain-of-function mutation of the IFL1/REV gene, is associated with alterations in the polarity of leaves, stems and carpels Plant and Cell Physiology 2004 45 369 385 10.1093/pcp/pch051 15111711

[B14] Ohashi-Ito K Demura T Fukuda H Promotion of transcript accumulation of novel *Zinnia *immature xylem-specific HD-Zip III homeobox genes by brassinosteroids Plant and Cell Physiology 2002 43 1146 1153 10.1093/pcp/pcf135 12407194

[B15] McConnell JR Emery J Eshed Y Bao N Bowman J Barton MK Role of PHABULOSA and PHAVOLUTA in determining radial patterning in shoots Nature 2001 411 709 713 10.1038/35079635 11395776

[B16] Baima S Nobili F Sessa G Lucchetti S Ruberti I Morelli G The expression of the Athb-8 homeobox gene is restricted to provascular cells in *Arabidopsis thaliana* Development 1995 121 4171 4182 8575317 10.1242/dev.121.12.4171

[B17] Baima S Possenti M Matteucci A Wisman E Altamura MM Ruberti I Morelli G The *Arabidopsis *ATHB-8 HD-Zip protein acts as a differentiation-promoting transcription factor of the vascular meristems Plant Physiology 2001 126 643 655 10.1104/pp.126.2.643 11402194 PMC111156

[B18] Prigge MJ Otsuga D Alonso JM Ecker JR Drews GN Clark SE Class III homeodomain-leucine zipper gene family members have overlapping, antagonistic, and distinct roles in *Arabidopsis *development Plant Cell 2005 17 61 76 10.1105/tpc.104.026161 15598805 PMC544490

[B19] Emery JF Floyd SK Alvarez J Eshed Y Hawker NP Izhaki A Baum SF Bowman JL Radial patterning of *Arabidopsis *shoots by class III HD-ZIP and KANADI genes Current Biology 2003 13 1768 1774 10.1016/j.cub.2003.09.035 14561401

[B20] Cronk QCB Plant eco-devo: the potential of poplar as a model organism New Phytologist 2005 166 39 48 10.1111/j.1469-8137.2005.01369.x 15760349

[B21] Floyd SK Zalewski CS Bowman JL Evolution of class III homeodomain-leucine zipper genes in streptophytes Genetics 2006 173 373 388 10.1534/genetics.105.054239 16489224 PMC1461458

[B22] Prigge MJ Clark SE Evolution of the class III HD-Zip gene family in land plants Evolution & Development 2006 8 350 361 10.1111/j.1525-142X.2006.00107.x 16805899

[B23] Degroote DK Larson PR Correlations between net auxin and secondary xylem development in young *Populus-deltoides* Physiologia Plantarum 1984 60 459 466 10.1111/j.1399-3054.1984.tb04912.x

[B24] Ingouff M Farbos I Lagercrantz U von Arnold S PaHB1 is an evolutionary conserved HD-GL2 homeobox gene expressed in the protoderm during Norway spruce embryo development Genesis 2001 30 220 230 10.1002/gene.1068 11536428

[B25] Namroud M-C Guillet-Claude C MacKay J Isabel N Bousquet J Molecular evolution of five regulatory genes in the conifer *Picea*: evidence for selection and large-scale demographic changes Molecular Evolution 2010 in press 10.1007/s00239-010-9335-1 PMC2874021 20354847

[B26] Tuskan GA DiFazio S Jansson S Bohlmann J Grigoriev I Hellsten U Putnam N Ralph S Rombauts S Salamov A Schein J Sterck L Aerts A Bhalerao RR Bhalerao RP Blaudez D Boerjan W Brun A Brunner A Busov V Campbell M Carlson J Chalot M Chapman J Chen GL Cooper D Coutinho PM Couturier J Covert S Cronk Q Cunningham R Davis J Degroeve S Dejardin A Depamphilis C Detter J Dirks B Dubchak I Duplessis S Ehlting J Ellis B Gendler K Goodstein D Gribskov M Grimwood J Groover A Gunter L Hamberger B Heinze B Helariutta Y Henrissat B Holligan D Holt R Huang W Islam-Faridi N Jones S Jones-Rhoades M Jorgensen R Joshi C Kangasjarvi J Karlsson J Kelleher C Kirkpatrick R Kirst M Kohler A Kalluri U Larimer F Leebens-Mack J Leple JC Locascio P Lou Y Lucas S Martin F Montanini B Napoli C Nelson DR Nelson C Nieminen K Nilsson O Pereda V Peter G Philippe R Pilate G Poliakov A Razumovskaya J Richardson P Rinaldi C Ritland K Rouze P Ryaboy D Schmutz J Schrader J Segerman B Shin H Siddiqui A Sterky F Terry A Tsai CJ Uberbacher E Unneberg P Vahala J Wall K Wessler S Yang G Yin T Douglas C Marra M Sandberg G Van de Peer Y Rokhsar D The genome of black cottonwood, *Populus trichocarpa *(Torr. & Gray) Science 2006 313 1596 1604 10.1126/science.1128691 16973872

[B27] Chan RL Gago GM Palena CM Gonzalez DH Homeoboxes in plant development Biochimica & Biophysica Acta-Gene Structure and Expression 1998 1442 1 19 10.1016/s0167-4781(98)00119-5 9767075

[B28] Saitou N Nei M The neighbor-joining method: A new method for reconstructing phylogenetic trees Molecular Biology & Evolution 1987 4 406 425 10.1093/oxfordjournals.molbev.a040454 3447015

[B29] Jansson J and Douglas CJ *Populus*: A model system for plant biology Annual Review of Plant Biology 2007 58 435 458 10.1146/annurev.arplant.58.032806.103956 17280524

[B30] Benjamini Y Hochberg Y Controlling the false discovery rate - a practical and powerful approach to multiple testing Journal of the Royal Statistical Society Series B-Methodological 1995 57 289 300

[B31] Efron B Tibshirani R Storey JD Tusher V Empirical Bayes analysis of a microarray experiment Journal of the American Statistical Association 2001 96 1151 1160 10.1198/016214501753382129

[B32] Rise ML Jones SRM Brown GD von Schalburg KR Davidson WS Koop BF Microarray analyses identify molecular biomarkers of Atlantic salmon macrophage and hematopoietic kidney response to *Piscirickettsia salmonis *infection Physiological Genomics 2004 20 21 35 10.1152/physiolgenomics.00036.2004 15454580

[B33] Morey JS Ryan JC Van Dolah FM Microarray validation: Factors influencing correlation between oligonucleotide microarrays and real-time PCR Biological Procedures Online 2006 8 175 193 10.1251/bpo126 17242735 PMC1779618

[B34] Whippo CW Hangarter RP A brassinosteroid-hypersensitive mutant of BAK1 indicates that a convergence of photomorphogenic and hormonal signaling modulates phototropism Plant Physiology 2005 139 448 457 10.1104/pp.105.064444 16126860 PMC1203393

[B35] Lafarguette F Leple JC Dejardin A Laurans F Costa G Lesage-Descauses MC Pilate G Poplar genes encoding fasciclin-like arabinogalactan proteins are highly expressed in tension wood New Phytologist 2004 164 107 121 10.1111/j.1469-8137.2004.01175.x 33873473

[B36] Espartero J Sanchez Aguayo I Pardo JM Molecular characterization of glyoxalase-I from a higher plant: Upregulation by stress Plant Molecular Biology 1995 29 1223 1233 10.1007/BF00020464 8616220

[B37] Galichet A Gruissem W Developmentally controlled farnesylation modulates AtNAP1; 1 functions in cell proliferation and cell expansion during *Arabidopsis *leaf development Plant Physiology 2006 142 1412 1426 10.1104/pp.106.088344 17041028 PMC1676069

[B38] Anterola AM Lewis NG Trends in lignin modification: a comprehensive analysis of the effects of genetic manipulations/mutations on lignification and vascular integrity Phytochemistry 2002 61 221 294 10.1016/S0031-9422(02)00211-X 12359514

[B39] Xiang S Usunow G Lange G Bush M Tong L Crystal structure of 1-deoxy-D-xylulose 5-phosphate synthase, a crucial enzyme for isoprenoids biosynthesis Journal of Biological Chemistry 2007 282 2676 2682 10.1074/jbc.M610235200 17135236

[B40] Loivamaki M Louis S Cinege G Zimmer I Fischbach RJ Schnitzler JP Circadian rhythms of isoprene biosynthesis in Grey poplar leaves Plant Physiology 2007 143 540 551 10.1104/pp.106.092759 17122071 PMC1761966

[B41] Sakakibara K Nishiyama T Kato M Hasebe M Isolation of homeodomain-leucine zipper genes from the moss *Physcomitrella patens *and the evolution of homeodomain-leucine zipper genes in land plants Molecular Biology and Evolution 2001 18 491 502 11264400 10.1093/oxfordjournals.molbev.a003828

[B42] Devos KM Brown JKM Bennetzen JL Genome size reduction through illegitimate recombination counteracts genome expansion in *Arabidopsis* Genome Research 2002 12 1075 1079 10.1101/gr.132102 12097344 PMC186626

[B43] Zhong RQ Ye ZH IFL1, a gene regulating interfascicular fibre differentiation in *Arabidopsis*, encodes a homeodomain-leucine zipper protein Plant Cell 1999 11 2139 2152 10.1105/tpc.11.11.2139 10559440 PMC144121

[B44] Otsuga D DeGuzman B Prigge MJ Drews GN Clark SE REVOLUTA regulates meristem initiation at lateral positions Plant Journal 2001 25 223 236 10.1046/j.1365-313x.2001.00959.x 11169198

[B45] Kim J Jung JH Reyes JL Kim YS Kim SY Chung KS Kim JA Lee M Lee Y Kim VN Chua NH Park CM microRNA-directed cleavage of ATHB15 mRNA regulates vascular development in *Arabidopsis *inflorescence stems Plant Journal 2005 42 84 94 10.1111/j.1365-313X.2005.02354.x 15773855 PMC1382282

[B46] Zhong RQ Taylor JJ Ye ZH Transformation of the collateral vascular bundles into amphivasal vascular bundles in an *Arabidopsis *mutant Plant Physiology 1999 120 53 64 10.1104/pp.120.1.53 10318683 PMC59269

[B47] Larson PR Isebrands JG The plastochron index as applied to developmental studies of cottonwood Canadian Journal of Forest Research 1971 1 1 11 10.1139/x71-001

[B48] Chang S Puryear J Cairney J A simple and effcient method for isolating RNA from pine trees Plant Molecular Biology Reporter 1993 11 113 116 10.1007/BF02670468

[B49] Kirst M Myburg AA De Leon JPG Kirst ME Scott J Sederoff R Coordinated genetic regulation of growth and lignin revealed by quantitative trait locus analysis of cDNA microarray data in an interspecific backcross of *Eucalyptus* Plant Physiology 2004 135 2368 2378 10.1104/pp.103.037960 15299141 PMC520804

[B50] Pavy N Paule C Parsons L Crow JA Morency MJ Cooke J Johnson JE Noumen E Guillet-Claude C Butterfield Y Barber S Yang G Liu J Stott J Kirkpatrick R Siddiqui A Holt R Marra M Seguin A Retzel E Bousquet J MacKay J Generation, annotation, analysis and database integration of 16,500 white spruce EST clusters BMC Genomics 2005 6 144 10.1186/1471-2164-6-144 16236172 PMC1277824

[B51] Chenna R H Sugawara T Koike R Lopez T Gibson TJ Higgins DG Thompson JD Multiple sequence alignment with the Clustal series of programs Nucleic Acids Research 2003 31 3497 3500 10.1093/nar/gkg500 12824352 PMC168907

[B52] Hall TA Hall N BioEdit: a user-friendly biological sequence alignment editor and analysis program for Windows 95/98/NT Nucleic Acids Symposium Series 1999 41 95 99

[B53] Kumar S Nei M Dudley J Tamura K MEGA: A biologist-centric software for evolutionary analysis of DNA and protein sequences Briefings in Bioinformatics 2008 9 299 306 10.1093/bib/bbn017 18417537 PMC2562624

[B54] Benson D A Karsch-Mizrachi I Lipman DJ Ostell J Wheeler DL GenBank Nucleic Acids Research 2005 33 D34 D38 10.1093/nar/gki063 15608212 PMC540017

[B55] Rozen S Skaletsky H Primer3 on the WWW for general users and for biologist programmers Methods in Molecular Biology 2003 132 365 386 10.1385/1-59259-192-2:365 10547847

[B56] Pulikowska J Twardowski T The elongation factor 1 from wheat germ: structural and functional properties Acta Biochimica Polonica 1982 29 245 258 7158172

[B57] Bedon F Grima-Pettenati J Mackay J Conifer R2R3-MYB transcription factors: sequence analyses and gene expression in wood-forming tissues of white spruce (*Picea glauca*) BMC Plant Biology 2007 7 7 17 10.1186/1471-2229-7-17 17397551 PMC1851958

[B58] Le Bail A Dittami SM de Franco PO Rousvoal S Cock MJ Tonon T Charrier B Normalisation genes for expression analyses in the brown alga model *Ectocarpus siliculosus* BMC Molecular Biology 2008 9 75 10.1186/1471-2199-9-75 18710525 PMC2546422

[B59] Christensen AH Sharrock RA Quail PH Maize polyubiquitin genes - Structure, thermal perturbation of expression and transcript splicing, and promoter activity following transfer to protoplasts by electroporation Plant Molecular Biology 1992 18 675 689 10.1007/BF00020010 1313711

[B60] Hellens RP Edwards EA Leyland NR Bean S Mullineaux PM pGreen: a versatile and flexible binary Ti vector for *Agrobacterium-*mediated plant transformation Plant Molecular Biology 2000 42 819 832 10.1023/A:1006496308160 10890530

[B61] Mukherjee K Burglin TR MEKHLA, a novel domain with similarity to PAS domains, is fused to plant homeodomain-leucine zipper III proteins Plant Physiology 2006 140 1142 1150 10.1104/pp.105.073833 16607028 PMC1435804

[B62] Leple JC Brasileiro ACM Michel MF Delmotte F Jouanin L Transgenic poplars - Expression of chimeric genes using 4 different constructs Plant Cell Reports 1992 11 137 141 10.1007/BF00232166 24213546

[B63] Franklin GL Preparation of thin sections of synthetic resins and wood-resin composites and a new macerating method for wood Nature 1945 155 51 10.1038/155051a0

[B64] Pitre FE Lafarguette F Boyle B Pavy N Caron S Dallaire N Poulin PL Ouellet M Morency MJ Wiebe N Lim EL Urbain A Mouille G Cooke JEK Mackay JJ High nitrogen fertilization and stem leaning have overlapping effects on wood formation in poplar but invoke largely distinct molecular pathways Tree Physiology 2010 20739427 10.1093/treephys/tpq073

[B65] Yang YH Dudoit S Luu P Lin DM Peng V Ngai J Speed TP Normalization for cDNA microarray data: a robust composite method addressing single and multiple slide systematic variation Nucleic Acids Research 2002 30 4 10.1093/nar/30.2.e4 PMC100354 11842121

[B66] Dudoit S Yang YH Callow MJ Speed TP Statistical methods for identifying differentially expressed genes in replicated cDNA microarray experiments Statistica Sinica 2002 12 111 139

